# Gamification in the classroom: Kahoot! As a tool for university teaching innovation

**DOI:** 10.3389/fpsyg.2024.1370084

**Published:** 2024-03-14

**Authors:** Agustín Aibar-Almazán, Yolanda Castellote-Caballero, María del Carmen Carcelén-Fraile, Yulieth Rivas-Campo, Ana María González-Martín

**Affiliations:** ^1^Department of Health Sciences, Faculty of Health Sciences, University of Jaén, Jaén, Spain; ^2^Department of Education and Psychology, Faculty of Social Sciences, University of Atlántico Medio, Las Palmas de Gran Canaria, Spain; ^3^Faculty of Human and Social Sciences, University of San Buenaventura-Cali, Cali, Colombia; ^4^Department of Psychology, Higher Education Center for Teaching and Educational Research, Madrid, Spain

**Keywords:** gamification, teaching innovation, university education, creative intelligence, concentration, attention, generic capabilities

## Abstract

**Objectives:**

The purpose of this study has been to evaluate the use of gamification in the classroom, in terms of its effects on attention, concentration, creativity, and generic capabilities, for university students enrolled in a Bachelor’s degree program in Physiotherapy.

**Methods:**

An experimental design was implemented, using three groups differentiated by their time of exposure to the game (0 min, 30 min, or 60 min per week). The sample consisted of 73 s-year students from a Bachelor’s degree program in Physiotherapy. The theoretical content for each class was taught during a period of 4 months, reinforced by use of the Kahoot! Online platform. Selective attention and concentration were evaluated using the d2 Test of Attention; creative intelligence using the Creative Intelligence Test (CREA); and generic capabilities using the capabilities subscale of the Student Engagement Questionnaire (SEQ).

**Results:**

The study’s participants had a mean age of 19.51 ± 0.9 years, and it has demonstrated that use of Kahoot! For longer periods of time, i.e., more than 60 min per day, can improve essential skills in university students, such as attention, creativity, critical thinking, self-managed learning, adaptability, problem solving, and computer literacy. This study’s results show that integrating Kahoot! Into the educational environment, especially with longer sessions that allow for deeper immersion in the game, produces benefits by stimulating various cognitive aspects and enhancing complex skills.

**Conclusion:**

This study has demonstrated that use of Kahoot! Improves key skills such as attention, creativity, and critical thinking, especially when longer sessions are used. It is also suggested that its use should be balanced with other educational activities, in order to achieve comprehensive development for the students.

## 1 Introduction

Teaching in the 21st century requires the use of active approaches that give students a prominent role during the learning process, and their educational experiences need to be useful and broadly applicable. This means that the students should be given opportunities for interaction, cooperation, competition, etc. (Martínez, 2017), because these aspects are typically associated with improved motivation during the teaching-learning process, as demonstrated by recent studies (Rodríguez et al., 2019; Darling-Hammond et al., 2020). New educational demands require new teaching strategies that can improve the dynamics of the learning process, and one of these new strategies is known as gamification (Li et al., 2023), whose benefits in the educational environment have been widely documented (Dahalan et al., 2023).

Gamification is defined as an educational strategy that adapts elements of game playing, so that they can be applied in the learning environment, with the aim of improving the students’ academic performance (Chen and Liang, 2022). Incorporating game-based elements can increase attention and engagement (Licorish et al., 2018), enhance motivation for working in groups, and in general, produce a more effective commitment to learning (Müller et al., 2015). It is an approach that can potentially enhance the learning process (Johns, 2015; Kalu and Bwalya, 2017), with an impact on the students’ grades and creation of a more satisfying educational experience (Ismail and Mohammad, 2017).

Integrating gamification into the educational environment requires strategic use of the mechanics offered by the games themselves, such as rewards, competition, levels, and goals, but in contexts unrelated to those games (Arnold, 2014). This approach also requires development of creative activities that can challenge the students to complete tasks in an innovative and original way (Lee and Hammer, 2011; Calvo and López-Rodríguez, 2021). In turn, this contributes to development of social skills, as the students are confronted by the challenges and objectives presented by the educational games (Villalustre and Del Moral, 2015). According to Kapp (2012) and Zichermann and Cunningham (2011), the aim of gamification is to make use of game-like strategies and mechanics to strengthen individual engagement and increase learning and motivation, among other objectives. It has been demonstrated that gamification can provide multiple advantages, with some of the highlights being the psychological benefits of improved concentration and attention (McGonigal, 2011), promotion of deductive and spatial thinking (Miller, 2013), and stimulation of imagination and creativity (De Soto, 2018). We agree with the conclusion reached by De Soto (Uyulgan and Akkuzu, 2004), that when an activity is enjoyable, the brain is able to assimilate information better. Gamification is therefore an approach that can help optimize learning, thereby improving the acquisition of knowledge (Ortiz et al., 2018).

There are currently a variety of online applications available on the market, which allow creation of interactive tests and questionnaires for use in the classroom, with the students able to participate by using their own mobile devices (Lozada and Gómez, 2017). These are tools that make it possible to develop games, activities, and questionnaires, and this can encourage more active student participation. The introduction of innovative teaching techniques, such as competition tests, motivates the students to play a more dynamic and interactive role (Orbegoso, 2016). The games are appealing to them, and by reviewing questionnaires or encouraging discussions during the game, these activities can become exciting and attractive experiences for the students (Oliva, 2017). In addition, the use of devices such as iClickers and smartphones, along with free online tools such as Socrative, Kahoot! And Quizizz, are making evaluation of students easier by providing real-time results. They also make it possible for instructors to obtain specific feedback, regarding the level of student comprehension of the contents they are teaching in the classroom (Pintor-Díaz, 2017).

After performing some in-depth background research and exploring various options, we decided to implement a methodology based on gamification by using the Kahoot! Platform. Our belief was that this would represent an opportunity to stimulate motivation, improve group dynamics, increase attention and critical thinking, and enhance learning among the students (McLaughlin and Yan, 2017). Kahoot! Is a type of software that allows the instructor to present questions or questionnaires related to the material being taught, with the students then able to respond using their own mobile devices, such as smartphones, tablets, or notebook computers (Cerro, 2015). The students can therefore use their mobile devices to review or apply what they have learned in an entertaining way, by merging games, learning, and new technologies during the educational process (Parra-González and Segura-Robles, 2019). In Spain, Kahoot! Has become one of the most popular entertainment-based digital learning tools in recent years, with the advantages of being free, easy to use, and effective in terms of improving classroom dynamics. It was conceived in 2013 by Professor Alf Inge Wang at the Norwegian University of Science and Technology, with the aim of generating enjoyable and functional educational environments (Martínez, 2017).

Kahoot! Is therefore a tool that can be incorporated into a teaching curriculum to enhance interdisciplinary skills, while also effectively integrating digital technologies and communications as resources for learning. It can stimulate motivation among the students, while also assisting with collection of data regarding their learning process and their understanding of the contents being taught (Amores-Valencia et al., 2022). It can also make a significant contribution to increasing the students’ active participation and commitment in the classroom, while fostering positive relationships among the various groups of students. In summary, all of this makes Kahoot! A valuable gamification tool for application during the educational process (Urh et al., 2015; Gokbulut, 2020; López-Belmonte et al., 2020), and it has already been used with success in a variety of university programs, to facilitate retention of complex theoretical concepts while at the same time stimulating the students’ intrinsic motivation to learn (Rodríguez, 2017).

In this context, the objective of our study is to evaluate the impact of gamification in the university educational environment, specifically among students enrolled in the Bachelor’s degree in Physiotherapy. We intend to determine how the integration of game elements, particularly through the Kahoot! Platform, influences attention, concentration, motivation, creativity, and the social skills of the students. We hypothesize that gamification not only improves these key dimensions of learning but also contributes to a greater overall satisfaction with the educational experience.

## 2 Materials and methods

### 2.1 Research design and participants

An experimental design was implemented using three groups, differentiated by their time of exposure to the game (0 min, 30 min, or 60 min per week). The sample consisted of 73 students enrolled in their second year of a Bachelor’s degree program in Physiotherapy. The following criteria were used to select the students eligible to participate: (i) enrollment in the second year of the Bachelor’s degree program in Physiotherapy; (ii) owning a smartphone; (iii) willingness to participate and signing an informed consent form. Before the study began, all eligible participants signed an informed consent form approved by the University of the Atlántico Medio’s Human Research Ethics Committee (CEI/01-007), which was designed to comply with the Declaration of Helsinki, best clinical practices, and the applicable laws and regulations. The study was conducted from September to December 2022, with registration number NCT06142812.

### 2.2 Intervention

The intervention carried out with Kahoot! Was meticulously planned to evaluate its effect on student learning over a four-month period. During this time, educational reinforcement sessions were organized using Kahoot! Divided into two intervention groups, differentiated by the duration of each session: one enjoyed 30-min sessions per week, while the other had 60-min sessions. A third group, which served as a control group, did not use Kahoot! For feedback, opting instead for conventional teaching methods with slide presentations.

Structured sessions with Kahoot! They were designed to consolidate the understanding of the theoretical concepts addressed in class. For this purpose, a specific questionnaire was developed in Kahoot! For each topic, composed of 15 questions, each with three answer options. These questionnaires were administered right at the end of the theoretical presentations of each unit, with the purpose of reinforcing and evaluating the students’ immediate understanding of the most recently taught content.

The dynamics of the sessions with Kahoot! Encouraged active participation: students entered the game through a unique PIN, using nicknames to maintain anonymity. The questions were projected on a large screen and accompanied by four visual options, along with a countdown timer, adding an element of gamification and friendly competition to the activity. Students selected the answer they considered correct using their mobile devices connected to the Internet. Immediately after answering, they received feedback on their choice, allowing the instructor to clarify doubts and explain the reasons behind the correct and incorrect answers.

This method of immediate feedback, added to the competitive and playful structure of the sessions, not only encouraged the attention and interest of the students, but also promoted an active and participatory learning environment. At the end of each questionnaire, a leaderboard was displayed with the names of the students who had obtained the five best scores, based on accuracy and speed of response, which added an additional motivating element for the participants (Parra-González and Segura-Robles, 2019). The possibility of downloading the results of the sessions allowed the instructor to identify both the questions that had presented the most difficulties to the students and those that required additional support, thus facilitating more personalized and effective teaching. Since the Kahoot! App is free and easy to use, both instructors and students have considered it to be an essential tool for enhancing classroom dynamics (Amores-Valencia et al., 2022).

### 2.3 Outcomes

As part of this study, data was collected at the beginning of the research and again after the intervention period had ended. Socio-demographic data related to the participants was also compiled, such as their age, sex, income level, and any learning difficulties.

To ensure validity, the chosen measures underwent extensive review by experts in the field to assess their appropriateness for the study population and research objectives. Additionally, a pilot test was conducted to evaluate the clarity and understandability of the measures for participants. Furthermore in this study, reliability was ensured through several methods. First, standardized procedures were followed consistently during data collection to minimize measurement error and variability. This included providing clear instructions to participants, using standardized assessment tools, and conducting assessments under similar conditions for all participants.

The Spanish version of the d2 Test of Attention (Seisdedos, 2012) can be used to evaluate selective attention and concentration in a classroom context. This test asks each participant to carefully check what is written on each line, from left to right, then cross out all instances of the letter “d” that have two small lines around them (two lines above, two lines below, or one above and one below). Those elements are considered relevant, while all other combinations (the letters “d” and “p” with other combinations of lines or no lines) are included as “distractors” and should not be crossed out. The participant is given 20 s to complete each line. In addition to demonstrating convergent and divergent validity, this test has been found to have excellent reliability, with both Cronbach’s alpha and test–retest reliability ranging between 0.90 and 0.97 (Pawlowski, 2020).

The Creative Intelligence Test (CREA) (Corbalán, 2006) evaluates creative intelligence by asking questions about an image. First, before the image is displayed, the participant fills in spaces using the information provided. The image is then displayed, and the participant’s task is to formulate as many questions as possible. Those questions are then assessed using specific guidelines, with positive scores given to appropriate questions, and questions that do not fit the context penalized. Additional points are awarded for compound questions. The final point score is calculated by taking into account the number of questions, omissions, canceled responses, and extra points. A high score (75–99 points) indicates excellent creative skills, a medium score (26–74 points) indicates a moderate level of creativity, and a low score (1–25 points) suggests limited creative capacity.

The student capabilities subscale of the Student Engagement Questionnaire (SEQ) developed by Kember and Leung (2009) and Gargallo et al. (2018) was also used to compile data regarding the students’ own reflections on their development of generic capabilities. This subscale contains 16 items that measure 8 aspects of capability: critical thinking (2 items), creative thinking (2 items), self-managed learning (2 items), adaptability (2 items), problem solving (2 items), communication skills (2 items), interpersonal skills and group work (2 items), and computer literacy (2 items). The participants’ responses are recorded using a 5-point Likert scale, ranging from 5 (“strongly agree”) to 1 (“strongly disagree”). The questionnaire also includes two open-ended questions to obtain comments about the best aspect and the aspect that most needs improvement. In this study, the capabilities subscale showed high reliability, with Cronbach’s alphas of 0.86 and 0.92.

#### 2.3.1 Sample size calculation.

Our study has been based on detecting a minimum clinically relevant change in the variables used to measure attention, creative intelligence, and SEQ capabilities. Using a power analysis, we determined that a sample size of 73 participants would give us a power of 80% for detecting these changes, using a 0.05 significance level and a realistic estimation of the effects based on previous research (Chan et al., 2021).

#### 2.3.2 Statistical analysis

An exploratory analysis was performed using the Kolmogorov–Smirnov test, to confirm the pre-training and post-training normality of the data. The results showed that the data had a normal distribution (*p* > 0.05), which justified the use of parametric tests. The pre-training and post-training values were presented as a mean and standard deviation, and a paired Student’s *t*-test was used to assess the statistical significance (*p* < 0.05) of the changes observed, using a stratified analysis based on time of exposure to the game. As part of the multivariate analysis of covariation (MANCOVA), the potential confusion effect caused by the variable that recorded the students’ sex was evaluated for all of the result variables, along with the interaction between the sex and time variables.

## 3 Results

The total sample consisted of 73 students with a mean age of 19.51 ± 0.95 years, all with attention difficulties, and with a higher proportion of male students (65.8%). Most of the students (76.7%) said that they enjoyed playing games on their mobile devices, with 39.7% reporting their daily use as between 1 and 60 min and the other 37% reporting more than 60 min of daily use (Table 1).

**Table 1:** Characteristics of the sample population.

	Mean		SD
Age	19.51		0.95
		*n*	%
Sex	Male	48	65.8%
	Female	25	34.2%
Like playing mobile games?	No	17	23.3%
	Yes	56	76.7%
Daily game minutes	0 min	17	23.3%
	1–60 min	29	39.7%
	> 60 min	27	37.0%

SD, Standard deviation.

A variety of tests were given to the students both before and after the intervention (Table 2). For the group that had no exposure to the game, there only significant differences observed were in the scores for adaptability (difference in means 4.42; *p* = 0.02), while those who played Kahoot! For 1–60 min each day showed differences only in their scores for adaptability (difference in means 29.66; *p* = 0.01) and computer literacy (44.48; *p* = 0.004).

**Table 2:** Pre- and post-intervention comparison for each form of evaluation, stratified by game time.

	0 min					1–60 min					> 60 min				
Variables evaluated	PRE		POST		*p*	PRE		POST		*p*	PRE		POST		*p*
	Mea*n*	SD	Mea*n*	SD		Mea*n*	SD	Mea*n*	SD		Mea*n*	SD	Mea*n*	SD	
d2	60.41	14.26	60.88	14.30	0.77	62.66	17.652	63.59	15.80	0.46	64.56	15.26	73.70	14.60	< 0.001
d2 Total	66.71	14.24	69.76	15.80	0.06	65.69	15.609	67.72	13.85	0.12	66.19	15.76	74.56	14.14	< 0.001
Creativity	57.88	18.30	56.88	18.43	0.55	58.83	21.899	57.76	22.46	0.46	56.48	18.44	64.48	17.19	< 0.001
Critical thinking	2.78	1.28	2.89	1.17	0.65	3.47	1.070	3.47	1.14	0.98	2.92	1.18	3.56	1.01	< 0.001
Creative thinking	2.84	960	2.74	963	0.70	2.87	0.995	2.76	0.84	0.54	2.88	1.12	3.35	1.05	< 0.001
Self-managed learning	3.55	955	3.33	760	0.39	3.39	0.809	21.82	97.36	0.32	3.40	0.95	4.26	0.67	< 0.001
Adaptability	3.19	789	3.63	838	0.02	3.45	0.806	3.75	0.84	0.01	3.60	0.74	4.30	0.65	< 0.001
Problem solving	2.94	1.07	3.25	1.03	0.19	3.11	0.872	3.43	0.97	0.06	3.20	1.12	04.02	0.80	< 0.001
Communication skills	3.30	951	3.19	1.08	0.51	03.09	0.783	3.14	0.92	0.68	3.17	1.13	3.12	1.06	0.589
Interpersonal skills and group work	3.22	937	3.13	970	0.58	03.09	0.914	2.95	0.96	0.22	3.00	1.17	2.95	1.14	0.658
Computer literacy	3.22	1.18	03.07	1.10	0.35	3.12	1.058	3.57	1.03	0.04	3.33	0.99	3.87	0.91	< 0.001

Finally, the students with more than 60 min of daily game playing showed improvement in most of the tests performed, with some of the most notable results being those for attention (difference in means 91.48), total attention (difference in means 83.70), creative intelligence (difference in means 80.00), critical thinking (difference in means 6.47), creative thinking (difference in means 4.70) self-managed learning (difference in means 8.57), adaptability (difference in mean 6.94), problem solving (difference in means 8.18), and computer literacy (difference in means 5.41), with these differences all significant (*p* < 0.001). However, there were no differences observed in relation to communication skills (*p* = 0.59) or interpersonal relationships and group work (*p* = 0.658). Furthermore, it is important to consider that, although students who played for more than 60 min daily showed significant improvements in a wide range of cognitive skills, we cannot disregard the influence of other external factors that may have contributed to these results. For instance, students’ personal commitment level to the game, their intrinsic motivation to participate, and their familiarity with the Kahoot! Platform could have influenced the magnitude and direction of the observed improvements. Likewise, students’ socio-economic and cultural background, as well as their prior educational level, might have played a role in their response to the game and in the acquisition of cognitive skills. Therefore, conducting more detailed analyses that take into account these potential confounding variables is essential for a comprehensive understanding of the effects of gaming on students’ cognitive development.

The multivariate analysis confirmed the significance of the differences seen when evaluating the group with the most game time exposure. The sex variable was discarded to eliminate any confusion effect, and the interaction between the game minutes and sex variables was also discarded, which indicates that regardless of a student’s sex, more than 60 min of game time can produce significant, beneficial differences, with the highest levels of impact on attention, creative thinking, adaptability, problem solving, and computer literacy (Wilks’ lambda = 1.99, *p* = 0.010) (Table 3).

**Table 3:** MANCOVA analysis adjusted for the daily game minutes variable and its interaction with the sex variable.

		*F*	*p*
Daily Minutes	Wilks’ lambda	1.99	00.10
	Dependent Variable	*F*	*p*
Daily minutes	d2	4.8292	0.011
	d2 Total	1.6176	0.206
	Creativity	1.0962	0.340
	Critical thinking	2.1384	0.125
	Creative thinking	3.3977	0.039
	Self-managed learning	7.417	0.480
	Adaptability	5.0949	0.001
	Problem solving	4.4251	0.015
	Communication skills	212	0.979
	Interpersonal skills and group work	2.089	0.812
	Computer literacy	3.3649	0.040
Sex	d2	5.304	0.469
	d2 Total	2.106	0.648
	Creativity	1.0335	0.313
	Critical thinking	0.550	0.815
	Creative thinking	4.918	0.486
	Self-managed learning	5.136	0.476
	Adaptability	0.680	0.795
	Problem solving	2.0778	0.154
	Communication skills	2.5373	0.116
	Interpersonal skills and group work	1.2383	0.270
	Computer literacy	2.1795	0.145
Daily minutes ✽ Sex	d2	2.1864	0.120
	d2 Total	6.082	0.547
	Creativity	2.0182	0.141
	Critical thinking	2.0488	0.137
	Creative thinking	1.6505	0.200
	Self-managed learning	3.710	0.691
	Adaptability	0.987	0.906
	Problem solving	3.232	0.725
	Communication skills	1.409	0.869
	Interpersonal skills and group work	1.3104	0.277
	Computer literacy	2.538	0.777

## 4 Discussion

The purpose of this research has been to evaluate the effects that use of the Kahoot! App on mobile devices may have on attention, creative intelligence, and the SEQ student capacities subscale, for students enrolled in their second year of the Bachelor’s degree program in Physiotherapy at the University of Jaén.

In our increasingly digital world, the use of digital tools in education has generated debates regarding their impact on student development (Martínez, 2017). Therefore, to help understand the potential role of technology in contemporary education, the present study has used differentiated groups of university students to address the question of whether regular use of the Kahoot! Platform, for more than 60 min per day, can improve essential skills such as attention, creativity, critical thinking, self-managed learning, adaptability, problem solving, and computer literacy.

In agreement with previous studies (Ismail and Mohammad, 2017; Licorish et al., 2018), our results support the conclusion that integrating platforms such as Kahoot! Into the educational process can produce multiple benefits. However, we should emphasize that the differences observed in our study are primarily seen in the group with the most time spent using that app (> 60 min per day), which would support the conclusion that the duration of use of such platforms may have a notable influence on the skills developed, because of various factors that may affect their impact. Use in the classroom for longer time periods allows deeper immersion in the dynamics of the game, which leads to development of a wider range of skills. By dedicating more time to use of the app, the students are faced with a greater diversity of challenges and situations that can stimulate various cognitive aspects (Rodríguez, 2017).

The complexity and variety of the challenges presented during longer usage sessions provide fertile ground for strengthening critical thinking and problem-solving skills. This is supported by previous research results demonstrating that additional usage time allows a more diverse set of problems to be incorporated, which in turn promotes development of more complex and sophisticated skills, and that prolonged use also allows for better adaptation to the game’s format and specific dynamics (Chacon and Janssen, 2020). In addition, longer periods of immersion may improve the students’ ability to adapt to other similar platforms and engage effectively with similar digital environments. Ongoing exposure to the Kahoot! Interface and mechanics during longer sessions may also increase computer literacy, which is supported by the observation that as students become more familiar with a platform’s features and characteristics, their competencies related to digital environments also improve (Wang and Thair, 2020).

With regard to specific skills, it has been demonstrated that in our modern context saturated with digital distractions, where attention has itself become an essential capability, the use of tools such as Kahoot! Can help students maintain their attention levels during longer sessions. This may be due to the fact that the platform’s interactive and competitive format encourages sustained concentration during educational tasks, offering an effective alternative to the distractions commonly existing in the digital environment (Aivaz and Teodorescu, 2022), and also the fact that prolonged engagement with challenging activities, such as interactive games, enhances attention (Fatima et al., 2019). In relation to this, the game’s interactive and challenging dynamics help improve concentration, mental resilience, and absorption of information, which are all important aspects of paying attention to details and increasing cognitive performance (Martinez et al., 2023).

Creative intelligence, as another essential aspect of student development, is stimulated by the entertaining nature of the Kahoot! Platform, because answering questions quickly, or creating questionnaires, inspires innovative thought in the participants and helps them generate original ideas. These effects have also been reported by Calvo and López-Rodríguez (2021), who found that spending longer time periods with activities such as those offered by Kahoot! Encourages exploration of diverse ideas and inspires creativity. In addition, the improvement seen in critical thinking skills may be due to the need to confront challenges presented in real time, because analyzing information, making quick decisions, and evaluating options contributes to the development of skills that are applicable not only in the academic world, but also in everyday life (Jin and Ji, 2021).

In addition, the results observed in this study regarding self-managed learning associated with the use of Kahoot! Can be attributed to the fact that it allows the players to advance at their own pace. This is also in agreement with previous research results that have emphasized independence as a driver of self-regulation and time management, which leads to continual improvement in terms of interaction with the contents being taught (Grabner-Hagen and Kingsley, 2023).

With regard to adaptability, our study indicates a clear association between exposure to the Kahoot! Platform and significant improvement in this skill among the students. This improvement was observed in all groups exposed to use of the game, although it was more pronounced among the students who used Kahoot! For more than 60 min each day. Given the critical importance of adapting to new contexts, both in the educational and work environments, prolonged immersion in Kahoot! Seems to facilitate better adjustment to the challenges presented during the game. This factor has potentially influenced the notable improvement seen in this skill, and has also been previously reported in the literature (Schmidt et al., 2011).

Problem solving is enhanced by the wide diversity of challenges presented when using Kahoot! The variety of situations and questions stimulates the students’ ability to quickly adapt to changing environments, and to find effective solutions in shorter periods of time (Kalleny, 2020). When the students dedicate more time to the game, they are able to experiment with different approaches to solving problems, which significantly contributes to their development of this skill. The game presents a wide range of situations that require adaptation to changing environments, and generation of effective answers in contexts where time is limited (Vlachopoulos and Makri, 2017). In addition, other researchers have reported that prolonged exposure facilitates experimentation with multiple strategies when solving problems, which also strengthens this skill (Donkin and Rasmussen, 2021; Teunisse et al., 2022).

With regard to self-managed learning, our study demonstrates that prolonged exposure to the game can be beneficial (Primack et al., 2012). This could be explained by the fact that the independence offered by Kahoot! Allows the players to advance at their own pace (Nieto-Escamez and Roldán-Tapia, 2021). The other studies included in a systematic review indicate that this independence facilitates more personalized interaction with the contents being taught, which has probably helped contribute to the improvement observed, and this can in turn stimulate self-regulation and time management (Edisherashvili et al., 2022).

Finally, computer literacy is strengthened by regular use of Kahoot! (Neureiter et al., 2020). Familiarity with digital interfaces and technological tools, and the ability to navigate smoothly through their contents, are important competencies in our increasingly digital world (Cortellazzo et al., 2019), and these are skills that can be strengthened through a deeper understanding of the platform’s features and characteristics. In contrast to previously reported results, however, we found that shorter sessions (from 1 to 60 min) tend to have effects focused on more immediate skills, such as adaptability and computer literacy. These shorter periods of interaction may not be sufficient to allow deeper exposure to more complex aspects involving critical thinking or creativity, which require more time and ongoing practice. It is also worth emphasizing that this study’s results did not show differences in communication skills or interpersonal relationships, regardless of the exposure time, which contrasts with the results reported by Chan et al. (2021), where the authors found differences in communication skills (*p* = 0.04) at the end of the course.

Another relevant limitation lies in the sample population, which is restricted to university students of a specific course at a particular institution. This limitation may restrict the generalizability of the findings to other student populations or educational contexts. Furthermore, the study design may not fully address the complexity of the effects of Kahoot use, as the specific learning context and individual characteristics of participants that could influence the results were not considered.

To address these limitations and advance the field of gamification in education, it is suggested to conduct more specific and targeted research. For example, future studies could consider including multiple educational institutions and educational levels to evaluate the transferability of the effects of Kahoot use in different contexts. Additionally, it would be beneficial to incorporate more detailed measures of individual participant characteristics, such as cognitive ability level, learning styles, and prior experience with technology, to better understand the factors that may modulate the developmental impact of Kahoot. Of skills. Likewise, it is suggested to explore the optimal duration and frequency of use of Kahoot to maximize educational benefits and avoid possible adverse effects, such as cognitive fatigue or loss of interest. These investigations could contribute significantly to the understanding of the role of gamification in education and provide guidance for effective and evidence-based educational practices.

In summary, ongoing use of Kahoot! During longer sessions could serve as a catalyst for development of critical skills in university students. However, it is essential to maintain a balance between the use of technology and other educational activities, to ensure comprehensive and diversified student development. In addition, the amount of time dedicated to technology-based activities may be a critical factor for allowing deeper, better consolidated development. This is because longer periods of use provide opportunities for more significant improvement, by giving students more time for in-depth exploration, reflection, and perfection of their capabilities.

## 5 Conclusion

The research reported here has demonstrated that use of the Kahoot! Platform for more than 60 min per day can produce significant results in terms of improving specific skills. This use for longer periods of time has revealed notable benefits in terms of attention, creativity, critical thinking, adaptability, problem solving, and computer literacy, therefore supporting integration of Kahoot! Into the educational process at universities. Our data suggest that inclusion of these digital activities for longer time periods promotes development of complex skills, and represents an effective means of cognitive enhancement and acquisition of essential skills. However, it is also worth emphasizing the need for a suitable balance between the use of technology and other educational activities, to ensure comprehensive and diversified student development. Ongoing use of these platforms provides opportunities for deeper, more exhaustive improvement of cognitive skills, which highlights the importance of dedicating sufficient time to activities of this type, to allow full development of the students’ capabilities.

## Data availability statement

The raw data supporting the conclusions of this article will be made available by the authors, without undue reservation.

## Ethics statement

The studies involving humans were approved by the Research Ethics Committee of the University of the Atlántico Medio (CEI/01-007). The studies were conducted in accordance with local legislation and institutional requirements. The participants provided their written informed consent to participate in this study.

## Author contributions

AA-A: Conceptualization, Methodology, Writing – original draft. YC-C: Conceptualization, Supervision, Writing – review & editing. MC-F: Methodology, Supervision, Writing – original draft. YR-C: Formal analysis, Writing – review & editing. AG-M: Formal analysis, Supervision, Writing – review & editing.
